# Child ViReal Support Program: A Randomized Controlled Trial Study for Effective Support of Parents Raising Children with Attention Deficits

**DOI:** 10.3390/bs13080691

**Published:** 2023-08-19

**Authors:** Iouliani Pachiti, Fotios S. Milienos, Panagiota Dimitropoulou

**Affiliations:** 1 Department of Psychology, University of Crete, 74150 Rethymno, Greece; psyp221@psy.soc.uoc.gr; 2 Applied Psychology Laboratory, Center for Research and Studies, University of Crete, 74150 Rethymno, Greece; 3 Department of Sociology, Panteion University of Social and Political Sciences, 17671 Athens, Greece; milienos@panteion.gr

**Keywords:** attention deficits, ADHD, multi-level interventions, RCT, psychoeducation, parent training, parenting stress, parental self-efficacy, parenting styles

## Abstract

Attention deficit hyperactivity disorder (ADHD) results in various functioning impairments in children’s lives and families. Parents of children with ADHD report high levels of parenting stress, low levels of parental self-efficacy, and use of more authoritarian and/or permissive parenting practices than parents of typically developing children. Intervention programs need to address both children’s and parents’ needs and multimodal intervention programs could cover this demand. The aim of this study was to examine the efficacy of “Child ViReal Support Program”—a multi-level evidence-based comprehensive program—on parenting stress, parental self-efficacy, parenting practices, and the core symptoms of children’s ADHD. Families with a child diagnosed with ADHD (*n* = 16) were randomly allocated to two groups (PC and CP; P = parent training, C = child training), and a cross-over design was utilized. Participating parents completed, in four different times during the study, the Parenting Stress Index-Short Form, the Parenting Sense of Competence Scale, the Parenting Styles and Dimensions Questionnaire-Short Version, and the parent form of the ADHD Rating Scale-IV. Parents from both groups, after their participation in the parent training, demonstrated reduced parenting stress, enhanced parental self-efficacy, and increased the employ of democratic parenting practices. More than that, they reported decreased levels of inattention and impulsivity/hyperactivity for their children. Evidence-based multi-level intervention programs could produce positive effects on parents and children by incorporating effective methods and tools in accordance with the needs and the demands of the family context.

## 1. Introduction

Attention, a multifaceted cognitive ability, plays a decisive role in various domains of human functioning, including cognitive development, social interactions, and academic endeavors [1,2]. For optimum performance in these domains, it is essential to develop and enhance attention skills, including selecting relevant information, task-focused concentration, and inhibiting impulsive responses [3]. However, a large proportion of school-aged children experience attention deficits that are often associated with neurodevelopmental disorders such as attention deficit hyperactivity disorder (ADHD) and can significantly impact their daily lives and overall functioning [4,5]. Hence, children with attention deficits experience impairments in neuropsychological functioning (e.g., executive functions) [3,6,7], emotional dysregulation (e.g., difficulty in the recognition and regulation of emotions, emotional negativity, etc.) [8,9], academic difficulties (e.g., poor academic performance, etc.) [10], adversities in their social/peer relationships (e.g., difficulties in forming and maintaining relationships, etc.) [11,12], and degraded quality of family life [13,14].

The challenges associated with ADHD and attention deficits extend beyond the affected child, causing severe dysfunctions to the entire family system. Parents of children with ADHD often experience increased parenting stress and reduced parental self-efficacy, which could negatively affect their well-being and the parent–child relationship [15,16]. Parenting stress, defined as “the aversive psychological reaction to the demands of being a parent” [17], is determined by child and parent characteristics and the surrounding relationship dynamics [18,19]. Parental self-efficacy, an individual’s confidence about their ability to successfully raise children, contributes significantly in perceiving and resolving parenting challenges [20,21].

Quantitative and qualitative research consistently reveals that parents of children with ADHD experience higher levels of related stress compared to parents of typically developing children [15,16,22,23] and/or parents raising children with other neurodevelopmental disorders [15]. Parenting stress often arises when parents perceive that the demands of their role exceed their resources for dealing with them [24]. Elevated parenting stress has been linked to various negative outcomes, including worsening of ADHD symptoms in children, reduced response to intervention, strained parent–child relationships, and decreased parental well-being [25,26]. Moreover, parents of children with ADHD often face adversities concerning family functioning and quality of life [13,14]. Thus, dysfunctional family dynamics coexisting with undermined daily life routines have been observed among parents and siblings of children with ADHD [13,14]. 

As far as parenting style is concerned, inadequate activity monitoring, an increase in punitive parenting techniques, and a decrease in supportive parenting practices are potentially signs of the negative impact of parenting stress on children with ADHD. Findings suggest a link between elevated parenting stress and more authoritarian and permissive parenting practices (for parenting styles’ description refer to Baumrind [27]), as well as difficulties with behavior regulation in children [22]. Also, lower levels of parental self-efficacy are linked to coercive parent–child interactions [28]. These unfavorable rearing practices have strongly correlated to exaggerated behavioral problems in children with ADHD [29] and poorer child executive functioning [22]. 

Considering the aforementioned challenges, several variables are highlighted in literature to mediate and/or moderate the impact of ADHD on parenting stress. Family impact, parental ADHD symptoms, the severity of a child’s symptoms, co-occurring emotional and behavioral difficulties, as well as contextual factors such as poor marriage quality and a lack of social support, have been included in relevant research [25,30,31,32].

Given the substantial impact of ADHD on children and family dynamics, the necessity to provide effective support and multifaceted interventions on the family system level seems to be impending. Pharmacotherapy, psychosocial interventions (such as behavior parent training, child training based on cognitive-behavioral therapy), and a combination of both are the most commonly implemented treatments for children with ADHD [33,34,35,36]. Pharmacological interventions have shown powerful short-term effects in reducing the core symptoms of ADHD [33,37], but these effects tend to diminish upon discontinuation [38,39,40]. Moreover, children need to be trained in skills that will help them with social and emotional functioning, whereas parents need to also be trained to be able to support their children and enhance their mental health.

In particular, the psychoeducation of parents raising ADHD children constitutes a systematic and didactic approach, providing substantial information on the disorder and its treatment, such as a detailed description of ADHD and its symptoms, informative knowledge about the epidemiology and etiology of ADHD, education on the available interventions, and other data [41,42,43]. On psychoeducation-based interventions, parents are regularly trained in behavioral strategies along with their children, so as to gradually build a common understanding of the disorder, evolve their skills, and be actively engaged in the comprehensive management of ADHD [42,43,44,45]. It is especially essential for mothers who seem to be more vulnerable, related to caregiving of children with ADHD and are at risk for depression and elevated stress [46], to participate in parent training programs. Hence, as far as psychosocial interventions are considered, they seem to lead to changes in inattention and behavioral difficulties often observed by children with ADHD [47,48,49], whereas they have positive effects on parents and family functioning [34,50]. Research indicates that parents report significant improvements in parenting stress and parental self-efficacy after participating in programs [44,51,52]. Likewise, their increased parenting sense of competence and decreased parenting stress after parent training have been associated with behavioral improvements in their children [52,53]. Also, parents’ participation leads to improvements in their parenting practices [50], their parent–child relationship, and the impact of child behavior on the family system [44,49,50]. 

Taking into consideration the aforementioned results and benefits of psychosocial interventions for children with ADHD and their parents, the “Child ViReal Support Program”—a comprehensive multi-level intervention program for children with attention deficits and their parents—was designed. This program combines parent training and child training, which utilizes the potential of immersive virtual reality (iVR) technology for the training of children’s attention. In the present study and manuscript, we mainly focus on the parent training component of the intervention program, and we assessed the impact of the intervention “Child ViReal Support Program” specifically on parenting stress, parental self-efficacy and parenting practices, as well as on the core symptoms of children’s ADHD (inattention, hyperactivity/impulsivity). The study addressed the following hypotheses: (1) after completing the intervention program (Time 3), parents would report decreased levels of parenting stress compared to their initial assessment (Time 1); (2) after completing the intervention program (Time 3), parents would report increased levels of parental self-efficacy compared to their initial assessment (Time 1); (3) parents would report decreased inattention and hyperactivity/impulsivity symptoms for their children at the end of the intervention program (Time 3) compared to their initial reports (Time 1); (4) after completing the intervention program (Time 3), parents would use more democratic parenting practices and less authoritarian and/or permissive parenting practices; (5) parents participating in the PC group, who completed the parent training before the child training, would report lower levels of parenting stress and higher levels of parental self-efficacy compared to parents who were randomized in the CP group (child training before parent training) at Time 2 assessment; (6) parents would report lower levels of parenting stress and higher levels of parental self-efficacy at the follow-up assessment, four months after the end of the intervention program (Time 4), compared to their initial assessment (Time 1); (7) at the follow-up assessment (Time 4), children’s inattention and hyperactivity/impulsivity symptoms would be significantly reduced compared to the initial assessment (Time 1); and (8) parents would use more democratic and less authoritarian and/or permissive parenting practices at the follow-up assessment (Time 4) compared to their initial assessment (Time 1).

## 2. Materials and Methods

### 2.1. Trial Design

A randomized controlled trial (RCT) study with a cross-over design was performed. Families were randomly assigned to either the PC group or the CP group (P = Parent training; C = Child training). Thus, families assigned to the PC group started with the parent training and continued with the child training, whereas families assigned to the CP group started with the child training and continued with the parent training. The study was registered in the Clinical Trials Registry (NCT05391698) [54].

### 2.2. Eligibility Criteria for Participants

Families were included in the study if (a) the child had a formal diagnosis of ADHD, (b) the child had Full-Scale IQ equal to or above 80 measured by the Wechsler Intelligence Scale for Children-Fifth Edition (WISC-V) [55], (c) the parents and the child could understand and speak fluently the Greek language, (d) the child had not been diagnosed with a comorbid disorder or other concurrent difficulties that may interfere with task performance during the assessments or the intervention program (e.g., autism spectrum disorder, pervasive developmental disorder, visual or hearing impairment, and psychotic disorder), (e) the child or the parents had not previously participated in an intervention program based on the behavioral or cognitive-behavioral approach, and (f) the child was not under any medication treatment for ADHD. 

### 2.3. Participant Recruitment

For recruiting families in the study, the research team contacted the experts and staff of the Interdisciplinary Evaluation, Counselling and Support Centers (KE.D.A.S.Y.) of Heraklio and Rethymno in Crete, Greece. The objectives of the study were explained to the staff of the centers, who then proceeded to inform the parents of children diagnosed with ADD/ADHD about the study. Families interested in participating could contact the research team via phone call or by filling out a participation form on the program’s website (https://sites.google.com/view/childvireal/home), which was created by the research team for the purposes of the study (accessed on 8 November 2021). Those families that fulfilled the screening inclusion criteria for the study (a, c, d, e, and f) were contacted by a research team member to schedule an appointment for the first assessment. Prior to participating in the study, both parents and children of the selected families provided separate written informed consent. During this 2 hrs appointment, parents provided the researchers with a copy of their child’s clinical assessment report supporting a primary clinical diagnosis of their child’s disorder. They also completed questionnaires about sociodemographic variables, child’s characteristics, diseases and comorbid problems, and family and/or other contextual factors. Regarding children, the WISC-V was performed to assess Full-Scale IQ [55]. Families that satisfied all the inclusion criteria for the study were contacted by the research team to participate in the “Child ViReal Support Program”.

The study was conducted in accordance with the Consolidated Standards of Reporting Trials (CONSORT) guidelines [56].

### 2.4. Ethics

The approval of the study was preceded by the Research Ethics Committee of the University of Crete (REC-UOC) (Approval Reference no. 51/25.02.2020). All participants had access to the information sheet and an online website for the study, which they were able to work through at their own pace. Furthermore, consent was obtained again after the first appointment as already mentioned.

### 2.5. Intervention Procedure

The comprehensive multi-level intervention program called the “Child ViReal Support Program” was designed by the research team and consists of a parent psychoeducational training program and a child training program. The main researcher developed both manuals of the training programs, noting in detail all the exercises and informative material that would be provided to participants. Both training programs were delivered by two licensed Educational/School Psychologists, who were recruited and trained in the use of the manuals. Systematic supervision sessions were delivered on a weekly basis by the designers of the intervention program, namely, a licensed Educational/School Psychologist, and an Assistant Professor in Educational Psychology. 

#### 2.5.1. Parent Psychoeducational Training Program

Parents attended an in-person manualized psychoeducational training program focused on ADHD. The program was based on well-evidence-based interventions developed by Barkley [42] and Kazdin [43], incorporating neuropsychological findings about the cognitive functions of children with attention deficits [57,58] and neurocognitive training [59,60]. The parent training was delivered in eight weekly group sessions (1.5–2 h per session), each focusing on a different thematic unit through lectures, group discussions, modeling, and role-playing. The first session provided an overview of ADHD diagnosis, including underlying neurobiology, associated executive function deficits, and behavioral difficulties faced by children with ADHD at home and school context. Subsequent sessions addressed parenting stress management techniques, behavior modification principles, effective communication techniques, and the design and implementation of a contingency management system. The fifth and sixth sessions focused on various executive functions (e.g., working memory, cognitive flexibility, emotional control, etc.) as well as strategies that parents could utilize to help their children concentrate, organize, remember, and learn more effectively. The seventh session was focused on social skills and emotional regulation and provided parents with techniques to support their children, respectively. The eighth session was a wrap-up of the program, summarizing the main points of all previous meetings. Also, parents had to complete weekly homework assignments to facilitate the implementation of the newly learned skills at home. An overview of the sessions and thematic units covered in each session is presented in Appendix A.

Each of the two licensed Educational/School Psychologists delivered the parent training program in two groups: for the parents allocated to the PC group and the parents in the CP group. To maintain treatment fidelity, the psychologists covered the parent training program by conducting sessions following the manual. They were thoroughly trained, reviewed the topics to be covered before each session, and checked the coverage after the session by completing an integrity checklist of the meeting. Any topic omitted from a session (e.g., due to lack of time) was presented in the following one. 

#### 2.5.2. Child Training Program

The child training program consisted of 16 individualized in-person sessions (1 h per session). The program utilized the potential of iVR technology for the training of focused and sustained attention and incorporated practices based on the cognitive-behavioral approach to foster behavioral and emotional self-regulation skills in children. During the program, children gained knowledge about the interconnection of emotions, thoughts, and behaviors. They received training in techniques aimed at transforming negative automatic thoughts into positive ones, as well as strategies for effective identification and expression of emotions and management of challenging emotions (e.g., anger and stress). In addition, the program included comprehensive training in a range of social skills, encompassing conversational initiation and maintenance, displaying polite interactions, and conflict resolution. Problem-solving techniques emphasizing skills such as goal setting, time management, and the breakdown of goals into manageable steps were also introduced. Lastly, the program emphasized the development of self-observation, self-guidance, and self-reinforcement techniques to equip children with the necessary tools for pursuing their goals effectively. 

#### 2.5.3. Randomization

**Figure 1:**
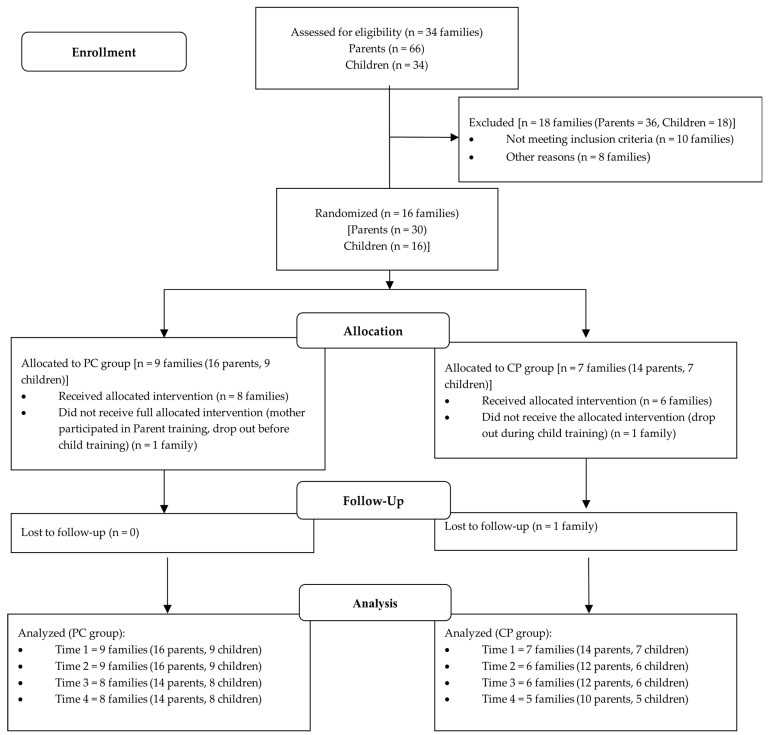
Participant flow diagram.

Sixteen families of children aged 9–12 years old (mean = 10.48, std = 0.94, 2 girls) with a diagnosis of ADHD participated in the study. Participants resided in the Heraklion and Rethymnon districts in Crete, Greece. As mentioned above, a randomized controlled trial (RCT) study with a cross-over design was performed. Therefore, using a web-based random number generator, families were allocated either to the PC group (*n* = 9) or to the CP group (*n* = 7). Two families dropped out of the intervention program for personal reasons. One family was assigned to the PC group, and the mother participated and finished the parent training program, but they dropped out after the Time 2 assessment. The second family was assigned to the CP group and dropped out during the child training (before the Time 2 assessment). A third family was not able to participate in the follow-up assessment, four months after the end of the intervention program (Time 4). The recruitment and randomization of families are illustrated in Figure 1. 

### 2.6. Measures and Procedure

#### 2.6.1. Primary Measures 

The measures of the RCT study are described below.

**ADHD Rating Scale-IV (ADHD-IV RS)** [61]. The ADHD-IV RS, which includes questionnaires for parents and teachers, is a tool for the early and accurate diagnosis of ADHD. For the purposes of the current study, the parent-report scale in its Greek adaptation [62] was used. This scale consists of 18 items addressing the different symptoms of ADHD as defined in the Diagnostic and Statistical Manual of Mental Disorders (DSM IV-TR) [63]. Hence, parents are asked to rate the frequency with which their child has demonstrated these symptoms at home over the previous six months (e.g., (Over the past 6 months) “Fails to give close attention to details or makes careless mistakes in schoolwork”, (Over the past 6 months) “Has difficulty playing or engaging in leisure activities quietly”). Three ratings/indicators are elicited by this scale: (1) the Inattention Score, (2) the Impulsivity/Hyperactivity Score, and (3) the Total Score. Based on the normative data of the Greek ADHD-IV RS [62], the internal consistency reliability for the three indicators (*a* = 0.84, *a* = 0.84, and *a* = 0.85, respectively) and the test–retest reliability (Pearson r index) over a four-week period (*r* = 0.78, *r* = 0.78, and *r* = 0.82, respectively) were in acceptable range. Regarding the discriminant validity, there were significant differences in the three scores between children with ADHD and children with generalized or specific learning difficulties. Also, the ADHD-IV RS has good predictive validity since the correlation between the parents’ scores and the diagnostic category ranged at medium levels, and the use of the parents’ scores led to a correct classification of about 76% of children [62]. 

**Parenting Stress Index-Short Form (PSI-SF)** [64,65]. The PSI-SF is a widely used and well-documented self-report tool for measuring parenting stress of parents of children 12 years and younger. Families identify the sources and different types of stress that comes with parenting such as children’s and parents’ characteristics, or different factors that are believed to affect their overall relationship. Thus, the PSI-SF provides an observation of parental stress levels as well as inadequate child rearing methods and a child’s adaptability in the family environment. Parents report their level of agreement with 36 items (e.g., “I feel trapped by my responsibilities as a parent”, “I feel that my child is very moody and easily upset”, etc.) using a 5-point Likert scale (1 = Strongly disagree, and 5 = Strongly agree). The scale consists of three subscales: Parental Distress, Parent–Child Dysfunctional Interaction, and Difficult Child in addition to a Defensive Responding Scale for an objective interpretation of parents’ responses and a Total Score. The internal consistency reliability (*a* = 0.91) and the test–retest reliability (*a* = 0.84) with a 6-month interval are in an acceptable range [64,65]. The PSI-SF questionnaire was translated and adapted into the Greek language by Leze [66].

**Parenting Sense of Competence Scale (PSOC)** [67,68]. The PSOC is a self-report scale for parents of children from birth to 17 years old. It consists of 17 statements that are assessed on a 6-point Likert-type scale (1 = Strongly disagree, and 6 = Strongly agree) and they relate to the parents’ sense and perceptions of their parenting ability and competence (e.g., “I honestly believe I have all the skills necessary to be a good parent to my child”), as well as their satisfaction with their parental role (e.g., “Considering how long I’ve been a parent, I feel thoroughly familiar with this role”). The PSOC extracts an Overall Parenting Competence Perception Score. According to various studies, the indices of internal consistency reliability for the Total Score range from 0.75 to 0.80, demonstrating that this scale possesses an acceptable level of reliability [68,69,70]. Also, according to the creators of PSOC [67,68], the content validity of the tool stems from the fact that it is based on a theory stating that parental self-confidence is composed of self-efficacy and satisfaction, which, in turn, regulates/moderates the parent–child relationship and their ability to effectively cope with the child’s challenging behavior. The criterion validity arises from the fact that research has shown a significant correlation between parental perceptions of children’s behavior and PSOC Total Score. Thus, parents of children who report more behavioral problems also report lower levels of parental self-esteem [68,70]. The scale was translated into the Greek language with the method of back-translation [71,72,73] by two independent bilingual psychologists, whose outcomes were identical to the original version. 

**Parenting Styles and Dimensions Questionnaire-Short Version (PSDQ-Short Version)** [74]. The PSDQ is a self-report questionnaire consisting of 32 statements that are assessed on a 5-point Likert scale (1 = Never, and 5 = Always). The PSDQ is based on Baumrind’s parental typology model [27] and assesses the typology of parents based on the parent–child relationship and communication, as well as on the practices applied by parents in the upbringing of their children. The questionnaire was adapted and standardized for the Greek population for fathers [75] and mothers [76] separately. The factor analysis applied to each questionnaire concluded in 29 statements (instead of 32), as three of them did not load on any factor and were removed. Four different factors (instead of three) emerged, which corresponded to four dimensions of paternal and maternal parenting styles, respectively. These dimensions concern the *democratic paternal and maternal styles* (13 items; *a* = 0.88 and *a* = 0.88, respectively) (e.g., “I allow my child to give input into family rules.”), the *authoritarian paternal and maternal styles* (7 items; *a* = 0.84 and *a* = 0.83, respectively) (e.g., “I explode in anger towards my child”), the *permissive paternal and maternal styles* (5 items; *a* = 0.63 and *a* = 0.65, respectively) (e.g., “I state punishments to my child and do not actually do them”), and the *strict paternal and maternal styles* (4 items; *a* = 0.70 and *a* = 0.68, respectively) (e.g., “I scold or criticize when my child’s behavior does not meet my expectations”). The strict parenting style combines elements and features of the democratic and authoritarian types but constitutes a separate parenting style. 

#### 2.6.2. Procedure

The participants underwent an initial baseline assessment (Time 1, T1), followed by random assignment to either the PC or CP groups. Subsequently, after completing the first phase of the intervention program (8 weeks), which entailed parent training for the PC group and child training for the CP group, the participants participated in a second assessment completing the same measures (Time 2, T2). Then, they proceeded to the second phase of the intervention program that involved child training and parent training, respectively. Upon the finalization of the intervention program (8 weeks), the participants were assessed again (Time 3, T3) with the same tools. Finally, a follow-up assessment was conducted four months after the intervention program’s conclusion (Time 4, T4), during which participants filled out the measures one last time. Summarizing, the time between baseline (T1) and Time 2 assessment was approximately 9 weeks, and between Time 2 and Time 3 was also approximately 9 weeks. The Time 4 assessment took place four months after the Time 3 phase was completed.

#### 2.6.3. Feasibility and Acceptability Measures

The research team, considering the important questions for the evaluation of an evidence-based intervention program, designed a questionnaire for the evaluation of the parent training program. The questionnaire has been created based on well-known questionnaires that have proven to be reliable and valid for assessing the social validity and satisfaction of an intervention program, such as the Therapy Attitude Inventory (TAI) [77,78], the Treatment Evaluation Inventory (TEI) [79], and the Program Evaluation Questionnaires for the Incredible Years intervention program [80].

**Evaluation of Parent Training—Final (EPT-F)**. After the parent psychoeducational training program, each participating parent completed the EPT-F questionnaire. The questionnaire is composed of 33 items, with 31 of them utilizing a 5-point Likert scale response format (1 = Strongly disagree, and 5 = Strongly agree) and two of them in the format of open-ended questions. The items assess the *feasibility* (e.g., “The knowledge gained, and skills developed through the program are useful for my relationship with my child”), *satisfaction* (e.g., “Overall, I feel satisfied with the program and its results”), and *practicality of the program* (e.g., “Practicing the exercises and newly acquired skills with my child at home proved to be useful”). The four sections of the EPT-F questionnaire are as follows: (1) Overall Program (9 items) that yields a Total Satisfaction Score, (2) Structure/Training (14 items) that provides a Program’s Usefulness Score, (3) Trainer’s Assessment (8 items) that produces a Trainer’s Assessment Score, and (4) Your Opinion (2 open-ended questions) in which parents could provide personal feedback regarding any aspects or features of the program they would like to alter, as well as their perceived main benefit from participating in the parent training program. 

### 2.7. Statistical Analysis

Statistical analysis of our dataset initially focused on descriptive statistics, computing the means, standard deviations, and correlations for each time point and group (PC and CP) separately; Friedman’s two-way analysis of variance was used for assessing the equality of means across time. The multivariate nature of such problems and the existence of longitudinal data (repeated measures on parents across the four time points) make the use of classical approaches, such as the ordinary multiple linear regression model, inappropriate due to the potentially correlated errors between measures of the same subject. Therefore, to deal with these issues, among others, we turn our attention to marginal models, one of the available procedures for such kind of data [81], focusing on the average effect of the independent variables (time, group, training participation, and parent’s gender and age) on our dependent variables (parenting stress, parental self-efficacy, and core symptoms of children’s ADHD). The data analysis was carried out using SPSS 28.0 [82] and r-project [83].

## 3. Results

### 3.1. Demographics

All children were diagnosed with ADHD by child psychiatrists at the Interdisciplinary Evaluation, Counselling and Support Centers (KE.D.A.S.Y.) and the Community Children and Adolescents’ Mental Health Centers of Heraklion and Rethymnon in Crete, Greece. The ages of the participating children ranged from 9 to 12 years old (mean = 10.48, std = 0.94), and the ages of the parents (14 fathers and 16 mothers) ranged from 32 to 52 years old (mean = 42, std = 5.11). Most participating parents had completed secondary education (*n* = 18, 60%), whereas some of them had a higher education degree (Bachelor = 9, and Master/Ph.D. = 2, 36.7%). Also, most parents were employed in full-time jobs (*n* = 23, 76.7%), whereas some were in part-time jobs (*n* = 3, 10%) or unemployed (*n* = 4, 13.3%). Out of 16 families, two were single-parent families. The majority of parents were living in urban areas (*n* = 19, 63.3%), whereas some resided in semi-urban areas (*n* = 5, 16.7%) and rural areas (*n* = 6, 20%). Most of the parents actively participating in the intervention program were female (14 mothers, and 1 father), and their mean age was 40.1 years old (the mean age for those not participating was 43.9).

### 3.2. Main Analysis

The descriptive statistics of the measures across the four time points of assessment (Time 1–4) and for each group separately (PC and CP) can be found in Table 1, along with Friedman’s two-way analysis of variance for assessing the statistical significance of the differences (the descriptive analysis and Friedman’s test were carried out by SPSS). Hence, referring to Parenting styles for the PC group, we can see that Authoritarian and Permissive styles keep decreasing, whereas the Strict style seems to be constant across time. Moreover, although the Democratic style seems to increase slightly from Time 1 to 2 (for the PC group), it remains constant afterward. This pattern is different in the CP group, where, in the first two time points, the Democratic style remains constant, but there is an increase from Time 2 to 3. As for the Authoritarian and Permissive styles (the styles with statistically significant differences across time, in the PC group), they both decrease as time passes; more precisely, significant differences seem to exist, according to the pairwise comparisons (adjusted by the Bonferroni correction). These results reveal that parents in general seem to adopt more democratic and less authoritarian and/or permissive parenting practices after their participation in the parent training program.

As far as Parenting Stress is considered, although a decreasing pattern across time seems to exist for all subscales, this pattern is more evident within the PC group than the CP group (apart from Parental Distress, all other subscales have statistically significant differences across time). The parental self-efficacy, as measured by the PSOC scale, is increasing as time passes; note the statistically significant differences between Time 1 and 4. 

Regarding the results from the ADHD-RS, the Inattention score for the PC group seems to keep decreasing from Time 1 to 3, but it remains constant from Time 3 to 4, whereas the Impulsivity/Hyperactivity score reveals a decreasing pattern in all time points (especially, after Time 3). As for the CP group, a more consistent decreasing pattern exists for both subscales (although the difference for Inattention is not significant). In general, all parents report fewer inattention and impulsivity/hyperactivity symptoms in their children after parents’ and/or children’s participation in the intervention program.

**Figure 2:**
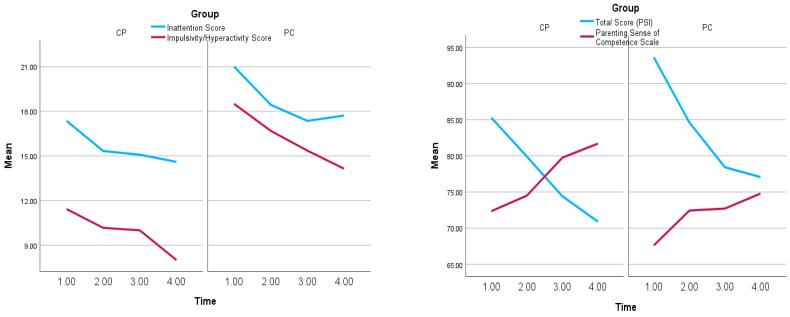
Mean plots of our dependent variables for each time and group (PC and CP), separately.

Figure 2 includes the means of parenting stress (Total Score), parental self-efficacy (parenting sense of competence; Total Score), and the two subscales of ADHD (Inattention and Impulsivity/Hyperactivity), across time and groups. Evidently, as hypothesized, all the variables are decreasing as time passes, except for parenting sense of competence, which is increasing. Note also that there is no evidence of interaction between time and group, i.e., the effect of time seems similar for each group. 

The correlations between the variables of our study, separately for each time point and group (PC and CP; the assessment of significance must be treated with caution due to the small sample sizes), were calculated (Supplementary Table S1). The correlation between Democratic parenting style and Parental Distress changes over time for the PC group, starting from positive at Time 1 and becoming negative. In contrast, for the CP group, the negative correlation between these variables decreases further as time passes. Also, the negative correlation between Democratic parenting style and Parent–Child Dysfunctional Interaction score at the beginning of the program (*r* = −0.292, *r* = −0.216 for Time 1 and 2, respectively) becomes much smaller after Time 2 for the PC group; for the CP group, there is an overall decrease from Time 1 to 4. The almost zero correlation between the Democratic parenting style and the Difficult Child subscale becomes negative as time passes for the PC group, but it seems to remain constant over time for the CP group. The same pattern seems to occur between the Democratic style and the Total Score of the PSI for the PC group. However, this is not the case for the CP group, where the negative correlation between the two variables is further decreasing as time passes. Additionally, the correlation between Democratic style and Impulsivity/Hyperactivity changes from strongly negative (*r* = −0.743) at Time 1 to almost zero (*r* = 0.079) at Time 4 for the CP group. On the other hand, there is a positive correlation between the Democratic style and the Total Score of the PSOC for both groups in each time point.

As far as the Authoritarian style and its correlations with the subscales of PSI are considered, there are positive correlations between this parenting style and the Parental Distress and Parent–Child Dysfunctional Interaction subscales for both groups (PC and CP). The negligible correlation between Authoritarian style and Difficult Child subscale becomes negative as time passes for the CP group. Reversely, the positive correlation between the Authoritarian style and Impulsivity/Hyperactivity at Time 1 for the CP group becomes negligible at Time 4. The same pattern occurs between the Authoritarian style and Inattention score for the PC group. Also, for both groups, there is a positive correlation between the Authoritarian style and the Total Score of parenting stress, whereas the correlation with parental self-efficacy is negative. 

The Permissive style seems to be more positively correlated with Parent–Child Dysfunctional Interaction, Difficult Child, and Total Score of PSI as time passes for both groups. In contrast, its correlation with Total Score of PSOC scale and Inattention is becoming negative, especially for the PC group.

Regarding the correlations of the scores from the ADHD-IV RS with the variables of PSI (Parental Distress, Parent–Child dysfunctional interaction, Difficult Child, and Total Score) for the PC group, Inattention shows a relatively small correlation with these variables at the first and last time points, but a moderate positive correlation at Time 2 and 3. Additionally, Impulsivity/Hyperactivity consistently correlates positively with PSI subscales over time for the PC group. In contrast, the CP group exhibits a change in correlation direction: the positive correlation between Inattention and Parental Distress at Time 1 becomes negative at Time 4, whereas the negative correlation between Inattention and Parent–Child Dysfunctional Interaction at Time 1 and 2 becomes positive at Time 3 and 4. Moreover, the positive correlation between Inattention and Difficult Child at the start of the study became even larger at Time 4 (from *r* = 0.406 to *r* = 0.841). Considering Impulsivity/Hyperactivity for the CP group, correlations with Parental Distress, Parent–Child Dysfunctional Interaction, and Total Score of PSI change from positive at Time 1 to negative at Time 4. 

As far as Parenting Sense of Competence is considered for the PC group, the negligible negative and positive correlation with Inattention at Time 1 and 4, respectively, are combined with moderate negative correlations at Time 2 and 3. Conversely, the negative correlation with Impulsivity/Hyperactivity at Time 1 increases by Time 4 (from *r* = −0.350 to *r* = −0.114). For the CP group, however, the negative correlation at Time 1 (*r* = −0.266) becomes positive by Time 4 (*r* = 0.176). 

The most important correlations, according to our aims, to be mentioned are the overall positive correlations of Democratic style with the parental self-efficacy (PSOC Total Score) over time, and the mainly negative correlations with the variables of PSI for the parents of both groups. In contrast, there are positive correlations between the Authoritarian and Permissive parenting styles with parenting stress and negative correlations with parental self-efficacy for both groups over time. 

Moreover, as one would expect, there are mainly positive correlations between the subscales of PSI across time for both groups. Conversely, there are mainly negative correlations between the parental self-efficacy (PSOC) with Parental Distress, Parent–Child Dysfunctional Interaction, and Total Score of PSI. As far as ADHD-IV RS is considered, there are mainly positive correlations between the two subscales (Inattention and Impulsivity/Hyperactivity) with Difficult Child and Total Score of PSI, whereas there are mainly negative correlations between the two subscales and PSOC for both groups across time. 

The next step would be the assessment of the effects of the intervention (time), group (“PC” and “CP”), training participation (“yes” and “no”), age, and parent’s gender on our dependent variables (parenting stress, parental self-efficacy, and core symptoms of children’s ADHD). However, although the correlation coefficients or the difference of means across groups or time provide us with useful perspectives in assessing the relationship between variables, they both ignore the multivariate nature of our data. Hence, we initially fit a set of different marginal models (due to the potentially correlated errors), according to the assumed structure of the covariance matrix of errors, and then the best model was given by the AIC and BIC criteria [84,85,86] (the function “gls” of “nlme” package in r-project was used, maximizing the restricted log-likelihood function with the generalized least squares method [87]). Two interaction terms were among the exploratory variables: between time and group, and between time and training participation.

Table 2 contains the estimated marginal models; however, following a backward selection procedure, all the interaction terms are excluded since their contributions are not significant. Thus, for each model, we resulted in keeping only time and group as exploratory variables. There is no evidence of a severe violation of model assumptions (i.e., linearity, normality of residuals, and independence between residuals and exploratory variables). All the assumptions have been checked based on the properties of the normalized residuals. Given the regression coefficients of Time, we can see that the estimations for parenting stress, Inattention, and Impulsivity/Hyperactivity decrease as time passes, (keeping the group fixed). Therefore, we expect a decrease of 5.31 units in stress, on average, from one time point to another. Also, we expect, on average, a decrease of 1.11 units in Inattention and 1.47 units in Impulsivity/Hyperactivity from one time point to another. In contrast, the effect of Time on parental self-efficacy is positive and equal to 3.10 units on average. 

Furthermore, the effect of the PC group on parental self-efficacy, as measured by the Total Score of the PSOC scale, and Impulsivity/Hyperactivity is significant. Thus, the estimation for the PC group for parental self-efficacy is lower than that of the CP group, by 5.94 units on average, whereas the estimation for Impulsivity/Hyperactivity is higher by 6.60 units on average (and keeping time fixed). 

Hence, as hypothesized, the results indicate that parents report lower levels of parenting stress and higher levels of parental self-efficacy following their participation in the intervention program compared to their initial (baseline) reports. In addition, as we predicted, parents report that their children’s inattention and impulsivity/hyperactivity symptoms have decreased since enrolling in the intervention program. 

### 3.3. Feasibility and Acceptability Outcomes

As previously stated, 14 mothers and 1 father with a mean age of 40.1 years old participated in the psychoeducational parent training. Over the course of the 8-week training, the participants attended 6.2 sessions on average (std = 0.77). In the final session, participants filled out the EPT-F questionnaire to rate their experience with the parent training. The descriptive statistics of the three scores that are extracted from the EPT-F (Total Satisfaction Score, Program’s Usefulness Score, and Trainers’ Assessment Score) and their correlation coefficients can be found in Table 3. It is worth mentioning that there were not any indications of correlations between the three scores of the evaluation of the program (EPT-F) and the dependent variables of our study. 

In general, participants were largely satisfied with the program (mean = 40.60, and std = 2.35). More specifically, the majority of parents (80% agree, and 13.3% strongly agree) reported that the skills they had learned in the program helped to mitigate their children’s behavior problems and difficulties, whereas only one parent was not sure (neither agree nor disagree). Furthermore, they all stated (60% agree, and 40% strongly agree) that they felt more confident in their ability to use the newly acquired skills to manage any future behavioral problems at home. When asked if their relationship with their child had improved since they began the program, the majority (73.3% agree, and 6.7% strongly agree) agreed, whereas three of them neither agreed nor disagreed. Additionally, they all consented that the program met their expectations (13.3% agree, and 86.7% strongly agree), and they would recommend it to parents with related issues (6.7% agree, and 93.3% strongly agree).

As far as the usefulness and feasibility of the program’s structure are concerned, participants found the program useful (mean = 63.80, and std = 4.24). More precisely, they all stated that the information offered in the program was understandable (26.7% agree, and 73.3% strongly agree) and beneficial (20% agree, and 80% strongly agree). Furthermore, most parents reported that using the newly acquired skills at home was simple (53.3% agree, 13.3% strongly agree, and 33.3% neither agree nor disagree) and useful (46.7% agree, 46.7% strongly agree, and 6.7% neither agree nor disagree). Even though some participants (two parents) expressed their desire for more sessions, the majority thought that the number of sessions was appropriate (40% agree, and 46.7% strongly agree). Moreover, all parents reported that the contents of the handouts given to them were concise and helpful, and they learned skills they intend to apply in the future. 

Regarding the evaluation of the facilitators (trainers) of the program, the participants highly rated their instructors’ competence, preparation, and effectiveness during the sessions. 

Taking into consideration the correlations between the three scores extracted from the EPT-F questionnaire, a high positive correlation between the parents’ perceived usefulness of the program and their overall satisfaction (*r* = 0.735, and *p* < 0.01) is revealed. Also, there is a positive correlation between the evaluation of the trainers with both their overall satisfaction from the program (*r* = 0.560, and *p* < 0.05) and their perceived usefulness of the program (*r* = 0.646, and *p* < 0.01). 

In addition to the quantitative data, the comments of the participants (qualitative feedback) for the parent training highlighted that they found the program of good quality, providing them with useful information about attention deficits and the co-occurring difficulties observed in children with ADHD. Also, they stated that the program contributed to organizing and comprehending their prior knowledge. More importantly, they acknowledged considerable progress on parenting skills and the acquisition of new techniques to interact and support their children effectively. They also pointed out that the psychoeducational training gave them the space to share experiences with other parents, which resulted in empowering each other to endure the adversities. Some of the parents’ insightful comments included the following: *“The main benefit I got [from the program] is that I apply better techniques to my child to help him improve a lot.”*, *“… I gained new knowledge and I shared similar experiences with parents of children facing related adversities.”, “The truth is that I see quite a big change in my child.”, “I am more capable of managing my child.”*, *“Definitely, I became a more effective parent and was helped to understand my child better”.*

## 4. Discussion

Attention deficits affect various domains in everyday lives of school-aged children that extend beyond the child’s struggles, harming well-being and disrupting the functioning of the entire family system [88,89]. Parents of children with ADHD encounter unique adversities in their parenting journey as they navigate the demands of managing their child’s symptoms and supporting their overall development [90]. In this effort, parenting stress emerges as a prominent concern for parents of children with ADHD. Recent studies consistently demonstrate significantly higher levels of stress experienced by these parents compared to parents of typically developing children [91,92]. The daily demands associated with managing the child’s behavioral difficulties, academic failures, and problematic social interactions contribute to elevated stress, adversely affecting parental well-being and influencing a parent–child relationship [14,30]. Additionally, parents of children with ADHD commonly report diminished levels of parental self-efficacy [93]. The negative consequences of elevated parenting stress and reduced parental self-efficacy affect the parenting practices used by the parents and the quality of parent–child interactions [22,94]. Addressing these challenges and providing effective support and intervention strategies for parents and children is crucial.

The current study examined the efficacy of the “Child ViReal Support Program”—a comprehensive multi-level intervention program for children with attention deficits and their parents—on parenting stress, parental self-efficacy, and parenting practices, as well as on the core symptoms of children’s ADHD (inattention and impulsivity/hyperactivity). In terms of parenting styles, the results indicate mainly negative correlations between democratic style and the subscales of PSI (Parental Distress, Parent–Child Dysfunctional Interaction, Difficult Child, and Total Score of parenting stress), whereas the correlation with parental self-efficacy was positive. In contrast, authoritarian and permissive parenting styles were positively correlated with parenting stress and negatively with parental self-efficacy. These results suggest that parents who tend to adopt a more democratic parenting style are likely to experience lower levels of stress in their role as parents, have healthier parent–child interactions, and perceive their child’s behavior as less challenging. They also tend to have higher levels of confidence and belief in their parenting abilities. Conversely, parents who adopt more authoritarian and/or permissive parenting practices are more likely to experience heightened stress levels in their parental role and may perceive themselves as less competent in their parenting abilities. These findings were in accordance with other research studies concluding that parents of children with ADHD tended to adopt more authoritarian and permissive parenting practices than parents of typically developing children [95], whereas the symptoms of children could predict the use of more controlling parenting [96,97]. Moreover, they underscore the importance of parenting styles in shaping parental stress and self-efficacy, and it seems crucial to consider these associations when developing interventions for parents of children with ADHD or other NDDs to promote positive parenting practices and mitigate parenting-related stress.

Furthermore, our results demonstrate a consistent decrease in authoritarian and permissive parenting styles among parents in both groups (PC and CP) over time. Conversely, there is a slight increase in the democratic style among parents in both groups, particularly from Time 1 to 2 for the PC group and from Time 2 to 3 for the CP group. Hence, it seems that parents, after participating in the training, managed to learn how to utilize more optimal parenting practices and engage in better interaction with their children. This finding was consistent with the results from the quantitative and qualitative feedback from the feasibility and acceptability measures of the parent training program, in which parents reported that they found the program useful and gained knowledge to understand their children better and be more effective in their roles. These results aligned with previous research indicating the effectiveness of parent training programs in promoting positive changes in parenting styles [50]. 

Also, the correlations between the core symptoms of ADHD (inattention and impulsivity/hyperactivity) and the variables of PSI reveal consistent associations. In general, there are positive correlations between the ADHD symptomatology and the subscales of PSI, indicating that higher levels of ADHD symptoms are associated with increased parental distress, dysfunctional interactions between parents and children, and difficulties in managing a challenging child. These findings are similar with other research studies showing that parents of children with ADHD report higher levels of parenting stress and that the severity of ADHD symptoms is positively related to parenting stress [25,30,98]. Conversely, the correlations between the two subscales of ADHD-RS and the PSOC are negative, a result which was also found in previous research [93]. These associations imply that there is a link between the presence of ADHD symptoms in children and how parents feel about their parenting abilities. Thus, as the severity of ADHD symptoms increases, parents may experience greater challenges in their parenting role leading to a lower perceived sense of competence. Conversely, when children exhibit fewer ADHD symptoms, parents may feel more capable and confident in their parenting skills.

Regarding parenting stress and parental self-efficacy, they seem to have a negative correlation across time. Research shows that their relations are reciprocal, with parents feeling more anxious if they feel less competent in their parenting role and vice versa [99]. Also, the results indicate that parents of both groups reported significantly lower parenting stress and enhanced levels of parental self-efficacy after their participation in the intervention program, and these changes remained significant at a follow-up assessment, four months after the end of the program. Previous research shows that participation in parent training programs has positive effects on parents since they feel more competent and less anxious at the end of the programs [29,100]. These findings are also congruent with the feedback given by parents at the end of the program, in which they reported that they felt more confident in their ability to manage their children’s behavior currently and in the future. 

As far as the core symptoms of ADHD are considered, parents observed fewer symptoms of inattention and impulsivity/hyperactivity in their children after their participation in the intervention program compared to their initial reports, and this decrease was present at a follow-up, namely, four months after the completion of the intervention. The decrease is evident in both symptoms for both groups starting from Time 2, which supports that both components of the intervention program (parent training and child training) have positive effects on the inattention and impulsivity/hyperactivity of the children. This is consistent with previous research demonstrating the efficacy of intervention programs for alleviating ADHD symptoms [50,101]. 

Furthermore, the parents of the PC group reported significantly lower levels of parental self-efficacy and higher levels of their children’s impulsivity/hyperactivity compared to the parents of the CP group. The impact of the group on these two variables might be explained by the difference observed in the initial assessment (at Time 1) between the two groups. Hence, it seems that parents of PC group observed higher impulsivity/hyperactivity symptoms in their children, which could affect their perceived efficacy on managing their children’s behavior (parental self-efficacy) since the severity of symptoms can have a negative effect on parents’ perceived competence. Another possible explanation for this finding, it could be that parents themselves have ADHD symptoms, which might result in lower levels of perceived efficacy in a variety of domains including their parenting role [93,102,103]. 

Overall, our study provides valuable evidence of the efficacy of the “Child ViReal Support Program” as an evidence-based intervention program for children with ADHD and their parents in improving parenting practices, diminishing parenting stress, enhancing parental self-efficacy, and reducing the core symptoms of ADHD. These findings are in accordance with prior research and highlight the importance of targeted interventions in supporting families affected by ADHD. 

However, it is crucial to acknowledge the limitations of the study, including the small sample size and potential biases associated with self-report measures. The sample size was small, especially when broken down into the two groups (PC and CP), resulting in decreased power to find significant effects between the two components of the intervention program (parent training and child training). In addition to this, the a priori differences between the PC and CP groups regarding the parental self-efficacy (lower in PC group) and the severity of ADHD symptoms (higher in PC group) indicate that the randomization of the families did not work out well enough. Nevertheless, it is of high importance and value that significant results were found even in this relatively small sample between the end of the intervention and the initial assessment of parents and children, indicating the necessity for follow-up research with larger samples. Another limitation of the study is the lack of information regarding parental ADHD symptoms, which could explain the differences observed in parental self-efficacy between the parents of the two groups. 

In conclusion, the “Child ViReal Support Program” demonstrates positive outcomes for children with ADHD and their parents in improving parenting practices and parental self-efficacy while reducing parenting stress and the core symptoms of ADHD. These findings have significant implications for practitioners (e.g., school psychologists), clinicians, and other professionals involved in supporting families dealing with ADHD. Also, the components of this program could be utilized in various contexts, whereas adaptations could be made to cater to children with other NDDs and their parents. The results extracted from the current study contribute to the growing body of literature on effective interventions for families dealing with ADHD, highlighting the importance of addressing both parent and child needs within evidence-based comprehensive treatment approaches. 

## Figures and Tables

**Table 1:** Means (std in parentheses) of subscales for each time point and group, along with Friedman’s two-way analysis of variance (testing equality of means across time).

Group	Scale	Subscale	Time 1 (*n* = 30)	Time 2 (*n* = 28)	Time 3 (*n* = 26)	Time 4 (*n* = 24)
PC	Parenting Styles and Dimensions Questionnaire	Democratic	4.03 (0.74)	4.16 (0.47)	4.16 (0.57)	4.15 (0.57)
		Authoritarian *	1.98 (0.51) ^1^	1.94 (0.52) ^1^	1.88 (0.61) ^1^	1.67 (0.49) ^1^
		Permissive **	3.11 (0.65) ^1^	3.09 (0.55) ^1^	3.03 (0.54) ^1,2^	2.66 (0.65) ^2^
		Strict	3.84 (0.52)	3.88 (0.48)	3.73 (0.50)	3.71 (0.59)
	Parenting Stress Index-Short Form (PSI-SF)	Parental Distress	29.25 (8.19)	26.25 (9.07)	25.07 (9.41)	25.93 (10.21)
		Parent–Child Dysfunctional Interaction **	29.25 (8.64) ^1^	25.88 (7.35) ^1,2^	25.29 (7.28) ^2^	23.14 (7.39) ^2^
		Difficult Child ***	35.13 (8.38) ^1^	32.50 (7.85) ^1^	28.07 (7.64) ^2^	28.00 (7.54) ^2^
		Total Score *	93.63 (22.70) ^1^	84.63 (20.61) ^1^	78.43 (21.69) ^1^	77.07 (22.43) ^1^
	Parenting Sense of Competence (PSOC)	Total Score *	67.63 (8.16) ^1^	72.44 (9.42) ^1,2^	72.71 (9.43) ^1,2^	74.79 (11.41) ^2^
	ADHD Rating Scale-IV	Inattention *	21.00 (3.37) ^1^	18.44 (4.27) ^1,2^	17.36 (4.27) ^2^	17.71 (4.81) ^1,2^
		Impulsivity/ Hyperactivity *	18.50 (5.09) ^1^	16.69 (6.02) ^1,2^	15.36 (4.77) ^2^	14.14 (5.78) ^2^
CP	Parenting Styles and Dimensions Questionnaire	Democratic *	4.18 (0.51) ^1^	4.15 (0.44) ^1^	4.38 (0.51) ^1^	4.30 (0.55) ^1^
		Authoritarian	1.64 (0.62)	1.67 (0.48)	1.40 (0.35)	1.47 (0.43)
		Permissive **	3.14 (0.49) ^1^	3.18 (0.40) ^1,2^	2.83 (0.61) ^1,2^	2.78 (0.69) ^2^
		Strict	3.55 (0.50)	3.50 (0.55)	3.19 (0.54)	3.45 (0.71)
	Parenting Stress Index-Short Form (PSI-SF)	Parental Distress	27.50 (6.60)	27.58 (6.43)	27.25 (6.45)	25.40 (7.52)
		Parent–Child Dysfunctional Interaction **	25.36 (6.83) ^1^	23.00 (4.67) ^1,2^	20.83 (5.80) ^2^	21.60 (5.72) ^2^
		Difficult Child ***	32.43 (5.50) ^1^	29.33 (8.04) ^1,2^	26.33 (6.44) ^2^	23.90 (5.69) ^2^
		Total Score **	85.29 (16.69) ^1^	79.92 (10.77) ^1,2^	74.42 (13.94) ^2^	70.90 (13.63) ^2^
	Parenting Sense of Competence (PSOC)	Total Score ***	72.36 (9.08) ^1^	74.50 (8.60) ^1^	79.75 (7.48) ^2^	81.70 (7.57) ^2^
	ADHD Rating Scale-IV	Inattention	17.36 (4.96)	15.33 (5.47)	15.08 (4.32)	14.60 (6.38)
		Impulsivity/ Hyperactivity *	11.43 (4.11) ^1^	10.17 (4.57) ^1,2^	10.00 (3.88) ^2^	8.00 (4.16) ^2^

^1,2^ means with the same or without superscripts have no statistically significant difference, according to the pairwise comparisons and Bonferroni adjustment using Friedman’s test. * *p* < 0.05, ** *p* < 0.01, and *** *p* < 0.001.

**Table 2:** Estimation of marginal models (std. errors in parentheses) for each of the four dependent variables (using generalized least squares estimation method).

	Dependent Variable			
Parameters	Parenting Stress	Parenting Sense of Competence	Inattention	Impulsivity/Hyperactivity
Intercept	91.54 (5.32)	70.01 (2.56)	18.42 (1.22)	13.05 (1.29)
Group (PC:1, CP:0)	5.56 (6.05)	−5.94 (2.99) *	2.84 (1.49)	6.60 (1.60) ***
Time (1–4)	−5.31 (1.29) ***	3.10 (0.57) ***	−1.11 (0.24) ***	−1.47 (0.23) ***
Correlation Structure	Autoregressive, AR (1)	Autoregressive, AR (1)	Compound symmetry	Compound symmetry

* *p* < 0.05 and *** *p* < 0.001.

**Table 3:** Means (std in parentheses) and Pearson correlation coefficients of the scores extracted from the Evaluation of Parent Training Final Questionnaire.

	Evaluation of Parent Training Final Questionnaire			
	Sessions Attended	Satisfaction Score	Program’s Usefulness Score	Trainers’ Assessment Score
Mean (std)	6.20 (0.77)	40.60 (2.35)	63.80 (4.24)	39.33 (2.05)
Correlations				
Satisfaction Score	0.439			
Program’s Usefulness Score	0.035	0.735 **		
Trainers’ Assessment Score	0.045	0.560 *	0.646 **	

* *p* < 0.05 and ** *p* < 0.01

## Data Availability

Data are available on reasonable request from the corresponding author.

## Supplementary Materials

The following supporting information can be downloaded at: https://www.mdpi.com/article/10.3390/bs13080691/s1, Table S1: Pearson correlation coefficients for each time point and group (PC and CP) separately.

## Appendix A

**Table A1:** Sessions of Parent Training Program.

Sessions	Content
Session 1	Attention deficits and concurrent difficulties Neurobiological background
Session 2	Parenting Stress Stress management strategies
Session 3	Positive attention and Praise Effective instructions/commands
Session 4	Contingency management system
Session 5	Executive functions I Strategies for the enhancement of executive functions
Session 6	Executive functions II Strategies for the enhancement of executive functions
Session 7	Emotional control Self-monitoring and self-regulation Strategies for the enhancement of children’s emotional control and self-regulation
Session 8	Wrap-up and review of the sessions and content covered through the parent training program
