# Supplementary material for: Child ViReal Support Program: A Randomized Controlled Trial Study for Effective Support of Parents Raising Children with Attention Deficits
Source: Behav Sci (Basel). 2023 Aug 19;13(8):691. doi: 10.3390/bs13080691 (PMC10451949; doi:10.3390/bs13080691)
Supplement: Supplementary file 1 [file behavsci-13-00691-s001.zip › behavsci-2508058-supplementary.pdf]

**Table S1.** Pearson correlation coefficients for each time point and group (PC and CP) separately													
			**PSDQ**			**PSI-SF**				**PSOC**	**ADHD-IV RS**		
	**Time**	**Group**	**Authoritarian**	**Permissive**	**Strict**	**Parental Distress**	**Parent-Child Dysfunctional Interaction**	**Difficult Child**	**Total Score**	**Total Score**	**Inattention Score**	**Impulsivity/ Hyperactivity Score**	**Total Score**
Democratic	1	PC	-.318	.267	-.225	.341	-.292	.038	.026	.627**	-.061	-.150	
		CP	-.916**	-.127	.443	-.334	-.520	-.100	-.378	.473	.253	-.743**	
	2	PC	-.300	-.098	.151	-.164	-.216	.021	-.141	.552*	.028	.001	
		CP	-.546	-.216	.199	-.332	-.412	.130	-.280	.530	-.216	-.224	
	3	PC	-.454	-.442	-.260	-.296	-.692**	-.465	-.524	.659*	-.216	-.390	
		CP	-.358	.191	.080	-.723**	-.404	-.075	-.537	.165	-.365	.102	
	4	PC	-.578*	-.594*	-.155	-.142	-.581*	-.326	-.366	.663**	.014	-.277	
		CP	-.716*	.063	.016	-.773**	-.725*	-.028	-.742*	.676*	-.043	.079	
Authoritarian	1	PC		.209	.544*	.512*	.611*	.394	.562*	-.711**	.267	.291	
		CP		.117	-.341	.349	.490	.058	.358	-.410	-.386	.671**	
	2	PC		.188	.129	.429	.535*	.380	.524*	-.589*	.484	.278	
		CP		-.086	.266	.672*	.212	-.282	.283	-.003	-.131	.233	
	3	PC		.594*	.599*	.675**	.527	.421	.618*	-.612*	.309	.246	
		CP		-.056	.345	.116	-.129	-.127	-.058	-.355	.069	.179	
	4	PC		.578*	.592*	.508	.689**	.582*	.654*	-.683**	.069	.211	
		CP		-.256	.536	.693*	.092	-.404	.252	-.561	-.337	.009	
Permissive	1	PC			.242	.366	-.001	.154	.189	.124	.024	-.099	
		CP			-.065	-.173	-.104	-.214	-.182	.113	-.004	.167	
	2	PC			.244	.342	.121	.005	.196	-.028	-.063	-.220	
		CP			-.499	-.067	.542	-.050	.158	-.522	-.452	-.361	
	3	PC			.101	.743**	.635*	.543*	.727**	-.600*	.156	.200	
		CP			-.659*	-.245	.165	.222	.058	.188	-.166	-.419	
	4	PC			.369	.356	.489	.387	.453	-.580*	-.290	.035	
		CP			-.722*	.088	.172	.045	.139	.041	-.058	-.217	
Strict	1	PC				.333	.429	.476	.459	-.323	.454	.538*	
		CP				-.125	-.460	.040	-.225	.819**	.348	-.180	

	2	PC		-.213	.315	.369	.159	.141	.157	.209
		CP		.179	-.105	.194	.206	.463	.195	.287
	3	PC		.333	.457	.449	.456	-.414	.307	.435
		CP		-.002	-.529	-.363	-.388	-.138	-.143	.258
	4	PC		.519	.555*	.556*	.606*	-.303	.173	.444
		CP		-.080	-.563	-.424	-.457	.143	-.159	.293
Parental Distress	1	PC			.648**	.745**	.882**	-.312	.099	.469
		CP			.685**	.727**	.915**	-.436	.260	.255
	2	PC			.510*	.475	.803**	-.640**	.472	.357
		CP			.318	-.421	.421	-.224	-.130	.160
	3	PC			.644*	.607*	.864**	-.765**	.302	.305
		CP			.198	.317	.692*	.062	.045	.141
	4	PC			.538*	.750**	.884**	-.743**	-.066	.235
		CP			.593	-.121	.750*	-.792**	-.365	-.323
Parent-Child Dysfunctional Interaction	1	PC				.758**	.894**	-.706**	.138	.483
		CP				.581*	.871**	-.435	-.163	.238
	2	PC				.793**	.883**	-.617*	.656**	.507*
		CP				.169	.750**	-.211	-.018	-.026
	3	PC				.854**	.916**	-.804**	.479	.551*
		CP				.488	.733**	-.127	.389	-.484
	4	PC				.814**	.848**	-.644*	.196	.369
		CP				.364	.899**	-.514	.230	-.121
Difficult Child	1	PC					.926**	-.416	.253	.651**
		CP					.855**	-.088	.406	.277
	2	PC					.872**	-.398	.768**	.703**
		CP					.568	.157	.316	.018
	3	PC					.902**	-.801**	.680**	.622*
		CP					.812**	.209	.110	.149
	4	PC					.946**	-.577*	.244	.577*
		CP					.503	-.161	.841**	.192
Total Score (PSI)	1	PC						-.535*	.181	.593*
		CP						-.379	.170	.290

	2	PC	-.653**	.734**	.606*
		CP	-.108	.150	.098
	3	PC	-.884**	.531	.536*
		CP	.073	.233	-.067
	4	PC	-.744**	.116	.423
		CP	-.719*	.246	-.149
Parenting Sense of Competence Scale (PSOC)	1	PC		-.034	-.350
		CP		.014	-.266
	2	PC		-.454	-.234
		CP		-.015	-.218
	3	PC		-.561*	-.463
		CP		-.452	-.275
	4	PC		.037	-.114
		CP		.140	.176
Inattention (ADHD-IV RS)	1	PC			.592*
		CP			.203
	2	PC			.721**
		CP			.733**
	3	PC			.760**
		CP			.179
	4	PC			.552*
		CP			.519
Impulsivity/ Hyperactivity (ADHD-IV RS)	1	PC			.592*
		CP			.203
	2	PC			.721**
		CP			.733**
	3	PC			.760**
		CP			.179
	4	PC			.552*
		CP			.519

*p<.05, **p<.01, (significance tests must be cautiously treated, due to small sample sizes).
